# Endocrine Actions of Osteocalcin

**DOI:** 10.1155/2013/846480

**Published:** 2013-04-30

**Authors:** Aurora Patti, Luigi Gennari, Daniela Merlotti, Francesco Dotta, Ranuccio Nuti

**Affiliations:** Department of Clinical, Surgical and Neurological Sciences, University of Siena, 53100 Siena, Italy

## Abstract

Osteocalcin is the most abundant noncollagenous protein of bone matrix. Once transcribed, this protein undergoes posttranslational modifications within osteoblastic cells before its secretion, including the carboxylation of three glutamic residues in glutamic acid, which is essential for hydroxyapatite binding and deposition in the extracellular matrix of bone. Recent provocative data from experimental observations in mice showed that the circulating undercarboxylated fraction of osteocalcin increases insulin secretion and sensitivity, lowers blood glucose, and decreases visceral fat in both genders, while it enhances testosterone production by the testes in males. Moreover, both total and undercarboxylated osteocalcins increase following physical activity with potential positive effects on glucose tolerance. Despite that these evidences have been only in part confirmed in humans, further prospective investigations are needed to definitively establish the endocrine role of osteocalcin both in the general population and cohorts of patients with diabetes or other metabolic disorders.

## 1. Introduction

**Figure 1:**
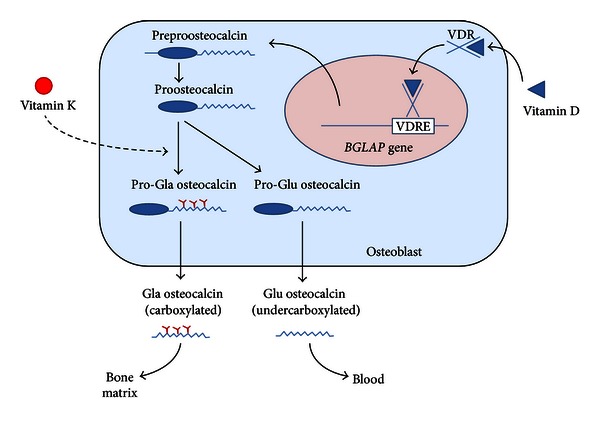
Osteocalcin synthesis in osteoblasts. The *BGLAP* gene encoding osteocalcin is mainly expressed in osteoblasts and to lesser extent odontoblasts. After transcription (which is stimulated by vitamin D) the preproosteocalcin peptide undergoes proteolysis giving rise to a prepeptide (23 aa) and a proosteocalcin peptide (75 aa). The latter can be carboxylated at Glu residues 17, 21, and 24, resulting in formation of Gla residues in a vitamin K dependent process. Generally, this process only occurs in a proportion of newly synthesized pro-osteocalcin. Then Gla and Glu pro-osteocalcin peptides are subjected to a final proteolytic process that produces, respectively, carboxylated and undercarboxylated osteocalcins. Both forms are released from osteoblasts in a process which is calciumdependent. While the carboxylated Glaresidues are involved in calcium and hydroxyapatite binding, allowing osteocalcin deposition on mineralized bone matrix, undercarboxylated osteocalcin has a low affinity for hydroxyapatite and is more easily released into the circulation.

Osteocalcin, also known as “*bone gamma-carboxyglutamic acid (Gla) protein* (BGP),” is the most abundant noncollagenous protein of bone matrix [1]. It is a product of differentiated osteoblasts, formed by 46 to 50 amino acids related to species [2–4]. The protein sequence is preserved in vertebrates especially in the central region that contains three residues of the amino acid gamma-carboxyglutamic acid (Gla). Once transcribed, osteocalcin undergoes posttranslational modifications within the osteoblast before its secretion. These include the proteolysis of a prepropeptide and the carboxylation of three glutamic residues (located in positions 17, 21, and 24) in glutamic acid [2] (Figure 1). Vitamin D stimulates directly osteocalcin transcription (in fact the gene has a “vitamin D responsive element”) while vitamin K regulates carboxylation processes. In addition, various growth factors, hormones, or cytokines can modulate osteocalcin production through signaling pathways or interacting with transcription factors that act on osteocalcin gene promoter region (*BGLAP* gene in chromosome 1q25–q31) [4]. This gene is generally inactivated during osteoblast proliferation, while it is abundantly transcribed during osteoblast differentiation. Carboxylated Glaresidues are involved in calcium and hydroxyapatite binding, allowing osteocalcin deposition in mineralized bone matrix [1]. On the contrary, noncarboxylated osteocalcin has a low affinity for hydroxyapatite and is more easily released into the circulation. However, both the carboxylated and the undercarboxylated forms are detectable in the peripheral blood, as well as total osteocalcin that is usually measured as a marker of bone formation. An immunoassay analysis in normal individuals estimated that up to 50% of osteocalcin is undercarboxylated and that this percentage may change in response to fluctuations in intakes of vitamin K on a daily basis [5, 6]. Thus, levels of undercarboxylated osteocalcin are influenced by vitamin K status, whereas total circulating concentrations of osteocalcin are influenced by bone cells activity independent of vitamin K [6].

Although osteocalcin is released by osteoblasts during bone formation and binds with the mineralized bone matrix [2], its precise function in bone metabolism has not been fully elucidated. Different experimental studies demonstrated that osteocalcin promotes the recruitment and differentiation of circulating monocytes and osteoclast precursors, suggesting its role on osteoblast-osteoclast interaction and bone resorption [2, 4, 7, 8]. Consistent with this observation, other studies have shown that osteoclasts poorly resorb bone areas which are deficient in osteocalcin [8]. Surprisingly and in contrast with these *in vitro* observations, the knock-out mice model for osteocalcin lacks negative skeletal abnormalities and shows higher bone mineral density without any change in bone resorption and mineralization [9].

Recently, however, experimental studies on mice models with osteoblast-specific overexpression or downregulation of osteocalcin production suggested that this protein might have an important endocrine function, outside bone, by regulating glucose and lipid homeostasis and probably also the production of testosterone by the testes [10–12].

## 2. Osteocalcin Effects on Glucose Homeostasis

### 2.1. Experimental Models

Most of information about the role of circulating osteocalcin and particularly its undercarboxylated fraction on energy expenditure and the regulation of insulin secretion was derived from studies on mice models in which osteocalcin production was inactivated or increased. In a first pivotal study aimed at identifying osteoblast-specific molecules that affect energy metabolism, Lee and colleagues [13] generated a knock-out mice model for the gene *Esp* (Esp^−/−^KO), which encodes for an extracellular tyrosine phosphatase named osteoarticular protein tyrosine phosphatase (OST-PTP), selectively expressed by osteoblasts, embryonic stem cells, and Sertoli cells [14, 15]. In bone this gene is upregulated positively during osteoblast differentiation and matrix deposition while it is downregulated in mineralizing osteoblasts. In this animal model, the lack of *Esp* gene was associated with high levels of adiponectin, undercarboxylated osteocalcin and also with impaired glucose metabolism [13]. In fact, after birth Esp^−/−^ mice developed hyperinsulinemic hypoglycemia with normal glucagon levels. This phenotype was essentially due to an increase in the number of pancreatic islets and beta-cell mass compared to control mice. Consistent with these observations, Esp^−/−^ mice showed increased tolerance during glucose tolerance test as well as increased insulin sensitivity with the insulin tolerance test as compared to the wild-type mice. The latter effect was probably to be ascribed, at least in part, to an increase in circulating adiponectin levels. Moreover, Esp^−/−^ mice showed increased energy expenditure, associated with a reduction in body fat and low levels of triglycerides and free fatty acids [13]. Notably, in this mice model osteocalcin gene expression was not altered, suggesting that the increase in the undercarboxylated form was independent of transcriptional mechanisms.

A further evidence on the endocrine action of osteocalcin was derived by experiments in mice in which the osteocalcin gene was deleted (Ocn^−/−^) [13]. These mice showed an opposite phenotype to that observed in Esp^−/−^ mice. In fact, they developed fasting hyperglycemia, hypoinsulinemia, insulin resistance, reduced energy expenditure, and obesity. The pancreas from these mice had a reduced number of islets and beta-cell mass, with decreased insulin production. Moreover, in addition to osteocalcin deficiency (genetically induced) both the expression of adiponectin by the adipose tissue and its circulating levels were significantly decreased compared to control animals. Finally, while the metabolic phenotype of Esp^−/−^ mice was fully corrected by removing one allele of osteocalcin, in Ocn^−/−^ mice a similar correction of phenotype was obtained with the intravenous administration of undercarboxylated osteocalcin [13]. Thus, in these models the variation in undercarboxylated osteocalcin levels was the main determinant of the observed alterations in glucose metabolism. In keeping with this observation, the administration of undercarboxylated osteocalcin was capable of preventing weight gain and the development of diabetes in normal mice receiving a hypercaloric and hyperlipidic diet [16].

Additional *in vitro* experiments showed that different concentrations of recombinant undercarboxylated osteocalcin are required to regulate insulin production by beta cells and adiponectin by fat cells [16]. While both beta-cell proliferation and expression of insulin gene were significantly affected by low concentrations of osteocalcin (between 6 and 60 pM), the production of adiponectin by adipocytes was stimulated at higher concentrations (between 0.6 and 6 nM). By contrast, the use of carboxylated osteocalcin did not produce any effect.

**Figure 2:**
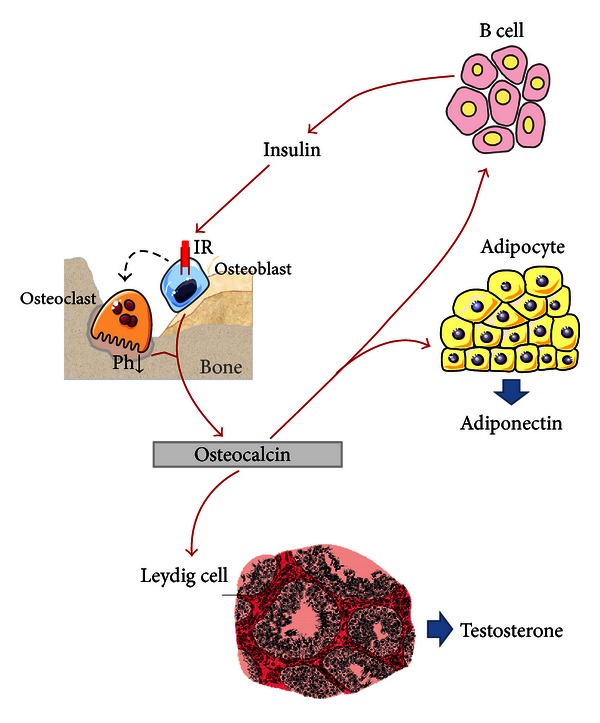
Endocrine actions of osteocalcin. Circulating osteocalcin and particularly its undercarboxylated fraction (released during active bone resorption) exert a direct effect on *β* cells, stimulating insulin production as well as on adipocytes enhancing adiponectin production. Adiponectin itself is able to promote insulin sensitivity. In turn, insulin also acts directly on osteoblast and indirectly on osteoclast. Osteoclast stimulates bone resorption with subsequent release of undercarboxylated osteocalcin in blood circulation. Finally, osteocalcin has a role also on Leydig cells, increasing their activity and testosterone production.

Overall, these experimental studies suggest an endocrine role of circulating undercarboxylated osteocalcin, with direct effects on insulin production by beta cells and indirect effects on insulin resistance, which are mediated by adiponectin secretion (Figure 2). Consequently, all conditions related to a reduced number or activity of osteoblasts should have a negative impact on glycemic control, as recently suggested by animal models [17]. In fact, the partial ablation of osteoblasts in mice produced negative effects not only on bone density and skeletal strength (as a result of reduced bone formation) but also on glucose metabolism leading to hyperglycemia, hypoinsulinemia, and insulin resistance similarly to what was observed in Ocn^−/−^ mice [17]. In addition, the administration of osteocalcin to these animals restored blood glucose and insulin levels, but with only a partial recovery of insulin sensitivity [17]. This suggests that osteoblasts exert a role on energy metabolism and on glycemic control through mechanisms dependent and independent of the production of osteocalcin.

As a parallel observation to the effect of osteocalcin on glucose metabolism, other experimental studies underlined a novel mechanism through which the pancreatic beta cells are able to regulate osteoblast activity and the release of undercarboxylated osteocalcin [18]. Indeed, osteoblasts express a functional insulin receptor (IR) and respond *in vitro* to physiological doses of insulin increasing their anabolic activities (through the production of alkaline phosphatase, osteocalcin, and the synthesis of collagen type one) and glucose uptake [18–21]. Moreover, more recent studies demonstrated that the selective deletion of IR in osteoblastic cells in mice (IRobs^−/−^) determined a reduction in the number and activity of these cells and was associated with skeletal abnormalities including a decrease in bone mineral density, especially at trabecular bone [22]. Together with a reduction in osteoblast number and activity there was a parallel decrease in osteoclast activity, with reduction in the depth of erosion lacunae and bone resorption, as evidenced by the decrease in bone resorption markers. In *in vitro* experiments, osteoblasts that did not express the IR showed a reduced proliferative capacity, were unable to fully differentiate in mature cells, and expressed reduced levels of osteocalcin [22]. This phenotype is remarkably consistent with clinical observations in diabetic patients which often show a decrease in bone turnover and particularly in markers of bone resorption as well as with experimental animal models of diabetes showing reduced osteoblast activity and decreased bone formation [23]. Surprisingly, in the IRobs^−/−^ mice, the lack of a functional IR in osteoblast was also associated with an increase in fat mass and a progressive impairment of glycemic control. This phenotype is reminiscent of the alterations observed in Ocn^−/−^ or Esp transgenic mice and was essentially due to a reduction in insulin secretory reserve in beta cells. In addition, total and undercarboxylated osteocalcin levels were significantly reduced in IRobs^−/−^ mice compared with controls. Therefore, the deletion of IR in osteoblasts leads to an impaired release of total and undercarboxylated osteocalcin from bone with negative effects on glucose tolerance. On the contrary, the selective deletion of IR in striated muscle cells or adipocytes did not exert any influence on glucose metabolism in mice models [24, 25].

The direct effect of insulin in osteoblasts and bone metabolism has been recently characterized in more detail using the IRobs^−/−^ and other mice models [26]. Overall, these experimental studies demonstrated that activation of insulin signaling in osteoblasts reduces the secretion of osteoprotegerin leading to an increase in osteoclast activity. In fact, osteoprotegerin is a decoy receptor for RANKL, a major cytokine involved in osteoclast differentiation and activation. The enhanced osteoclast activity following insulin signaling, in turn, promotes the acidification of the extracellular matrix of bone (since an acidic pH is essential for bone resorptive capacity) allowing protein decarboxylation and the release of undercarboxylated osteocalcin. Consistent with this mechanism, osteoprotegerin expression was increased while genes implicated in osteoclast activity and bone resorption were overexpressed in IRobs^−/−^ mice as compared with normal mice [26]. The opposite alterations were observed in Esp^−/−^ mice, likely due to the lack of inhibitory effect of this tyrosine phosphatase on insulin signaling in osteoblasts.

Therefore, together with the stimulation of osteoblast differentiation and bone formation, the effect of insulin in bone results in enhanced osteoclast activity, increased bone matrix acidification, and subsequent decarboxylation of osteocalcin, with a positive impact on glucose homeostasis. Notably, animal models of osteopetrosis due to *Tcirg1* gene mutation (encoding a vacuolar pump subunit which is essential for acidification of the bone matrix) showed an impaired glucose tolerance associated with reduced levels of undercarboxylated osteocalcin [26, 27]. Conversely, treatment with inhibitors of bone resorption such as alendronate reduced the levels of undercarboxylated osteocalcin and normalized the phenotype in Esp^−/−^ mice [26]. Thus, the acidification of the extracellular matrix of bone by active osteoclasts appears fundamental for osteocalcin decarboxylation and its effects on glucose metabolism in mice.

All the above experimental observations also indicated that the IR is substrate for the tyrosine phosphatase OST-PTP (encoded by *Esp *gene) and possibly PTP1B expressed by murine and human osteoblasts, respectively [26]. These tyrosine phosphatases may interfere with the carboxylation of osteocalcin through indirect mechanisms such as IR dephosphorylation and inactivation of insulin signaling in osteoblasts. In fact, in osteoblasts of Esp^−/−^ mice the phosphorylation of IR was increased, suggesting that the metabolic alterations in this animal model (mainly characterized by hypoglycemia and increased glucose tolerance) were secondary to an increase in insulin signaling in bone. In a similar manner, the phenotype of Esp^−/−^ mice was corrected by inactivation of an IR allele.

In summary, different experimental observations indicated the existence of a feedforward loop linking insulin, bone resorption, and osteocalcin activity as a potential mechanism for the association between bone and glucose metabolism. In support of these findings, the daily injection of osteocalcin in normal mice (in doses between 3 and 30 ng/g/day) led to an improved glucose homeostasis by increasing beta-cell function and insulin sensitivity [28]. Moreover, more recent studies in different mice models demonstrated that osteoblast-targeted disruption of glucocorticoid signaling (due to transgenic overexpression of the glucocorticoid-inactivating enzyme 11*β*-hydroxysteroid dehydrogenase type 2) significantly attenuated the suppression of osteocalcin synthesis and prevented the development of insulin resistance, glucose intolerance, and abnormal weight gain induced by corticosterone treatment [29]. These data suggested that the effects of exogenous high-dose glucocorticoids on insulin target tissues and energy metabolism may be mediated, at least in part, through the skeleton.

### 2.2. Clinical Evidences and Therapeutic Implications

The above experimental evidences have been only in part confirmed in humans and results remain conflicting. Indeed, the *Esp* gene is a “pseudogene" in man and does not play the same role demonstrated in mice [30]. Instead, in humans a similar role could be played by a different gene, named *PTP1B*, which encodes for a tyrosine phosphatase expressed in osteoblasts and is able to dephosphorylate the IR. Consistent with this hypothesis, a decrease of *PTP1B* gene expression in human osteoblasts increased IR and FoxO1 phosphorylation and decreased osteoprotegerin production [26]. Moreover, in keeping with the observations in osteopetrotic *Tcirg1* deficient mice, human cases of autosomal dominant osteopetrosis with mutations in *CLCN7* gene (encoding for a chloride channel coupled with the proton pump in osteoclasts) showed low circulating levels of undercarboxylated osteocalcin, associated with an increase in postprandial insulin [26]. A more recent study was designed to assess the effect of surgical resection on osteocalcin and blood glucose levels in patients with osteoid osteoma, a benign osteoblastic tumor, in comparison with subjects undergoing knee surgery or healthy individuals [31]. Of interest, the surgical resection of osteoid osteoma induced a consistent drop in total and undercarboxylated osteocalcin together with an increase in glucose levels, whereas the other bone remodeling parameters (CTX and bone alkaline phosphatase) were not affected. No significant variations were observed in controls. Thus, like in experimental models, at least in these rare human disorders, a consistent variation in osteocalcin production and/or decarboxylation could play a significant role on carbohydrate metabolism.

It is well known from clinical and experimental observations that markers of bone turnover including osteocalcin are lower in diabetic patients than in normal individuals and that interventions which improve glycemic control are generally associated with an increase in serum osteocalcin [6, 23, 32]. Conversely, whether an increase in osteocalcin levels is associated with a reciprocal improvement of glycemic control remains to be demonstrated from the clinical point of view. In fact, even though several studies in different populations of diabetic subjects or normal individuals have been performed in the last few years their results remain conflicting. Moreover, most of these reports were based on retrospective and cross-sectional analyses of studies that were not specifically designed to determine the relationship between osteocalcin and glucose metabolism. Despite these limitations, different cross-sectional and prospective studies in healthy subjects demonstrated a positive association between total osteocalcin levels and improved fasting blood glucose or reduced concentrations of glycosylated hemoglobin levels, even in different ethnic groups and at different ages [33–40]. In some of these studies high osteocalcin concentrations significantly correlated with an improved response to oral glucose load or the euglycemic clamp as well as with additional parameters of glucose tolerance such as the HOMA index, the index of Stumvoll, and the OGIS index [37, 38]. In addition, a recent prospective analysis carried out in middle-aged men showed that low osteocalcin levels were related to a high risk of developing type 2 diabetes at 10 years [41]. Similarly to what was observed in healthy populations, an inverse association between osteocalcin and glucose tolerance has been described also in some cohorts of patients with obesity, diabetes, metabolic syndrome, cardiovascular disease, and renal failure [42–49]. Moreover, a recent prospective study in patients with type 2 diabetes indicated that an increase in osteocalcin levels over a period of 6 months was associated not only with a decrease in glycosylated hemoglobin and an improved glucose tolerance but also with positive effects on triglycerides and HDL levels [41], suggesting that osteocalcin could exert a protective effect on cardiovascular risk, as further highlighted by other studies [50–52]. To this regard, however, other observations showed a positive rather than a negative correlation between total osteocalcin levels and cardiovascular risk [53, 54]. In one of these studies, in fact, elevated osteocalcin levels were associated with higher prevalence of carotid plaques, aortic calcification, and increased intima-media thickness [53]. This contrasts with the above evidences about a protective effect of osteocalcin on glucose homeostasis and metabolic syndrome, conditions which are also associated with increased cardiovascular risk. Indeed, in a recent prospective analysis on 3542 adult men aged between 70 and 90 years and followed for more than 5 years both lower and higher total osteocalcin levels predicted increased all-cause mortality rates, with comparable associations for cardiovascular and noncardiovascular deaths [55].

These discordant results may be related at least in part not only to the limited clinical significance of a single measurement of plasma osteocalcin or the potential influence of pharmacological treatment and lifestyle variables (i.e., nutritional status, smoking, or physical activity) but also to the fact that osteoblast-like cells have been identified in atherosclerotic lesions as well as in peripheral blood [56]. Thus, in some instances, part of circulating osteocalcin might directly reflect the degree of cardiovascular calcification and atherosclerosis instead of bone turnover. Moreover, the assessment of total osteocalcin does not completely account for the circulating undercarboxylated fraction, which has been shown to be the biologically active “hormone,” at least in animal models. Indeed, only few studies measured both the total and the undercarboxylated form of osteocalcin, which precludes definitive conclusions regarding the role of osteocalcin carboxylation in glucose metabolism. Overall, results from these studies were inconclusive [6, 32]. In fact both positive or inconsistent associations between undercarboxylated osteocalcin, glucose homeostasis, and the distribution of body fat were reported in population-based cohorts and in patients with diabetes or metabolic syndrome [57–65]. In addition, while in some of these studies the undercarboxylated form was better correlated with glycometabolic status than carboxylated or total osteocalcin levels, other studies did not show significant differences between the different osteocalcin fractions or even suggested a more consistent association with the measurement of total osteocalcin. A possible explanation for the lack of concordance among these studies could be in part related to the current limitations in the measurement of circulating undercarboxylated osteocalcin levels and the lack of standardization among the different techniques [66]. Undercarboxylated osteocalcin levels are also strictly associated with vitamin K status in contrast to total osteocalcin and surprisingly a high intake of vitamin K (resulting in a low proportion of undercarboxylated osteocalcin) has been associated with reduced insulin resistance, which is the opposite to what would be expected based on the above mouse models [6, 67]. Moreover, whereas in most animal species osteocalcin is fully carboxylated, in humans osteocalcin either in bone or serum is incompletely carboxylated, and the degree of carboxylation is determined by vitamin K availability in the diet [6]. Reduced circulating osteocalcin concentrations (up to 80%) were also observed in humans than in other species. Thus, caution needs to be used in the extrapolation of findings from the mouse models to humans.

It is well known that physical activity is able to exert an anabolic action on bone through the stimulation of bone formation, while inactivity is associated with an increase in bone resorption [68]. Accordingly, given the positive relationship between physical activity and the improvement of glucose homeostasis, a recent study investigated whether part of this interaction may be mediated by an increase in circulating osteocalcin levels [69]. To this aim 28 middle-aged obese men were randomly assigned to aerobic or power exercises. Of interest, the reduction in serum glucose after acute exercise (especially aerobic exercise) appeared at least in part related to increased undercarboxylated osteocalcin levels. Moreover, those subjects with higher baseline glucose and glycosylated hemoglobin had greater reductions in glucose levels after exercise. In particular, in the subgroup of obese men with type 2 diabetes undercarboxylated osteocalcin was among the strongest predictors for the change in glucose levels after exercise. These findings are in part consistent with the results of a previous study, that assessed the effects of resistance training in combination with dietary restriction for weight loss. Whereas an increase in osteocalcin levels was observed after training, these changes were significantly associated with changes in insulin resistance in the slight weight loss group but not in the moderate weight loss group [70]. However several other studies were not able to demonstrate a significant increase in osteocalcin levels with exercise and specifically with resistance training [6].

The effect of osteoclast activity and matrix acidification on the release of undercarboxylated osteocalcin from bone described in animal models, if proven in humans, might also have important clinical implications for the treatment of skeletal fragility in diabetic patients as well as in nondiabetic subjects. In fact, many of the drugs currently used for the treatment of osteoporosis are antiresorptives and consistently suppress osteoclast activity and bone resorption, with potential negative implications on bone matrix acidification and the release of undercarboxylated osteocalcin. Indeed, while initial clinical observations suggested that both total and undercarboxylated osteocalcin levels may be suppressed by antiresorptive treatment for osteoporosis [71, 72], a large retrospective analysis of postmenopausal women involved in randomized controlled trials with alendronate (a nitrogen containing bisphosphonate with potent antiresorptive activity on bone) failed to evidence any increase in diabetic risk in the treatment group as compared with place-treated subjects and even suggested a mild relative risk reduction with active treatment [73]. This latter observation has been confirmed by a more recent post hoc analysis of 3 placebo-controlled trials in postmenopausal women with antiresorptive agents (Fracture Intervention Trial of Alendronate, Health Outcomes and Reduced Incidence with Zoledronic Acid Once Yearly Pivotal Fracture Trial, and Fracture Reduction Evaluation of Denosumab in Osteoporosis Every 6 Months Trial) [74]. In all the 3 trials differences in fasting glucose changes from randomization to trial conclusion (3-4 years) between active treatment and placebo groups were not statistically significant. Moreover, diabetes incidence was not increased in any of the treatment groups or in the pooled estimate as compared with placebo. Similar effects were observed among the groups of overweight and obese women. A slight but significant increase in weight was observed with alendronate and denosumab treatment compared with placebo, and this increase was only in part accounted by the parallel gain in bone mass. However, despite a decrease in bone turnover markers was observed in these trials after treatment (consistent with the antiresorptive activity of bisphosphonates), neither total or undercarboxylated osteocalcin levels were measured. Conversely, a comparative analysis of osteoporotic women treated with anabolic (PTH 1-84) or antiresorptive (alendronate) compounds underlined potential differences in fat and energy metabolism between the 2 regimens [75]. In fact undercarboxylated osteocalcin levels increased significantly with PTH while they decreased with alendronate administration. Moreover, in the overall cohort, 3-month change in undercarboxylated osteocalcin was inversely associated with 12-month changes in body weight, fat mass, and adiponectin levels.

Even though these posthoc studies provided evidence that bone antiresorptive treatment for up to 4 years does not have major effects on glucose metabolism in postmenopausal women with osteoporosis, this information needs to be replicated on a prospective basis as well as in patients with diabetes or impaired glucose tolerance. In addition, given the reported association between vitamin D and glucose homeostasis [76, 77] a potential positive effect of vitamin D supplementation on the prevention of diabetes in either placebo and active treatment groups cannot be ruled out in these retrospective studies.

## 3. Effects of Osteocalcin on Testicular Function

As observed for energy metabolism, the demonstration that osteocalcin plays a biological function on the testes (through the regulation of androgen production) was derived from experimental studies on Esp^−/−^ and Ocn^−/−^ mice, characterized respectively, by excessive or absent osteocalcin production [78]. In fact, while female mice deficient in osteocalcin were fertile and did not show any gonadal abnormality, male mice with the same defect showed a poor reproductive activity, associated with a decreased volume of the testes, epididymis, and seminal vesicles. These alterations were also associated with a 50% decrease in sperm count. On the contrary, male Esp^−/−^ mice had an increase in testicular volume and a 30% increase in sperm count. None of these parameters were affected in mice with specific deletion of osteocalcin gene in Leydig cells only, suggesting that osteocalcin production by the skeleton is directly involved in endocrine regulation of male reproduction. Moreover, these mice models also demonstrated that osteocalcin deficiency impairs Leydig cells maturation and reduces testosterone synthesis. In fact, circulating androgen levels were significantly reduced in Ocn^−/−^ mice and increased in Esp^−/−^ animals. These observations were consistent with results in *ex vivo* cell assays demonstrating that a factor secreted by osteoblast (but not by other cells of mesodermal origin) is able to increase testosterone production in testis explant and primary Leydig cells but not in ovary explants [78]. In particular, in cultured Leyding cells deficient in osteocalcin, the treatment with supernatant of wild-type osteoblasts (containing osteocalcin) increased the maturation and the production of testosterone, while this effect did not occur with other cell lines. In the same studies, an unbiased approach based on the ability of osteocalcin to increase cAMP production in Leydig cells and on its dichotomy function between testis and ovary led to the identification of Gprc6a, an orphan transmembrane receptor coupled to G-proteins, as the potential osteocalcin receptor [78]. Of interest this receptor is expressed in human and mice Leydig cells of the testes but not in follicular ovarian cells, thus providing a molecular basis for the gender specific effect of osteocalcin on male fertility. Consistent with this hypothesis, the specific deletion of Gprc6a gene in Leydig cells decreased testosterone levels and male fertility in mice. Further analyses evidenced that by binding to Gprc6a osteocalcin regulates in a CREB-dependent manner the expression of enzymes required for testosterone biosynthesis in Leydig cells [78]. Overall these experimental evidences extend the endocrine role of osteocalcin, which may act not only as a regulator of energy metabolism in both genders but also of testosterone production and fertility in men (Figure 2).

A major question raised by these experimental observations is whether the skeleton also regulates testosterone production and male fertility in humans. Even though the available clinical information is limited, there have been 3 recent studies which seem to support the hypothesis of an endocrine role of osteocalcin on the male gonad in humans [79–81]. In the first study, carried out on young males during skeletal growth, osteocalcin levels significantly correlated with circulating testosterone [79], which was the major determinant of periosteal circumference as assessed through high-resolution pQCT performed on the radius. Similar correlations were demonstrated with the assessment of undercarboxylated osteocalcin. Moreover, the correlations between osteocalcin, testosterone, and periosteal circumference were highest in subjects with bone age between 11 and 14 years, during the phase of maximal skeletal growth. Thus, during this phase, the rise in osteocalcin levels (due to the rapid skeletal growth) may further stimulate testicular testosterone production, which, in turn, contributes to an increase in bone size. This could at least in part explain the fact that at the end of skeletal growth males show a larger bone size than females, despite a similar volumetric bone density [82]. In a different study, performed in a cohort of type 2 diabetic patients, circulating undercarboxylated osteocalcin positively correlated with free testosterone (even after correction for FSH and LH levels) and negatively with glycosylated hemoglobin levels [80]. Taken together, results from this study further confirmed the existence of a direct action of osteocalcin on testicular testosterone production in humans and provided a potential explanation for the low testosterone levels frequently described in diabetic subjects [83]. A similar correlation between osteocalcin and testosterone levels was observed in a population-based cohort of 1338 men (aged 25–86 years) independent of diabetes status as well as in a smaller sample of patients with bone disorders [81].

## 4. Conclusions

In summary the recent provocative investigations on animal models with excess or defect in osteocalcin production have introduced a new concept according to which the skeleton behaves as an endocrine organ by secreting osteocalcin which, in turn, leads to increased insulin secretion, lower blood glucose, increased insulin sensitivity, and decreased visceral fat in both genders and enhanced testosterone production by the testes in men. Although these evidences have been only in part confirmed in humans, further prospective investigations are needed to evaluate the clinical impact of variation in osteocalcin levels or its undercarboxylated fraction on endocrine functions both in the general population and cohorts of patients with diabetes or other metabolic disorders.

## References

[1] Razzaque MS (2011). Osteocalcin: a pivotal mediator or an innocent bystander in energy metabolism?. *Nephrology Dialysis Transplantation*.

[2] Hauschka PV, Lian JB, Cole DE, Gundberg CM (1989). Osteocalcin and matrix Gla protein: vitamin K-dependent proteins in bone. *Physiological Reviews*.

[3] Nielsen-Marsh CM, Richards MP, Hauschka PV (2005). Osteocalcin protein sequences of Neanderthals and modern primates. *Proceedings of the National Academy of Sciences of the United States of America*.

[4] Villafán-Bernal JR, Sánchez-Enríquez S, Muñoz-Valle JF (2011). Molecular modulation of osteocalcin and its relevance in diabetes. *International Journal of Molecular Medicine*.

[5] Booth SL, Rajabi AA (2008). Determinants of vitamin K status in humans. *Vitamins and Hormones*.

[6] Booth SL, Centi A, Smith SR, Gundberg C (2013). The role of osteocalcin in human glucose metabolism: marker or mediator?. *Nature Reviews Endocrinology*.

[7] Mundy GR, Poser JW (1983). Chemotactic activity of the *γ*-carboxyglutamic acid containing protein in bone. *Calcified Tissue International*.

[8] Glowacki J, Lian JB (1987). Impaired recruitment and differentiation of osteoclast progenitors by osteocalcin-deplete bone implants. *Cell Differentiation*.

[9] Ducy P, Desbois C, Boyce B (1996). Increased bone formation in osteocalcin-deficient mice. *Nature*.

[10] Ng KW (2011). Regulation of glucose metabolism and the skeleton. *Clinical Endocrinology*.

[11] Ducy P (2011). The role of osteocalcin in the endocrine cross-talk between bone remodeling and energy metabolism. *Diabetologia*.

[12] Karsenty G, Ferron M (2012). The contribution of bone to whole-organism physiology. *Nature*.

[13] Lee NK, Sowa H, Hinoi E (2007). Endocrine regulation of energy metabolism by the skeleton. *Cell*.

[14] Mauro LJ, Olmsted EA, Skrobacz BM, Mourey RJ, Davis AR, Dixon JE (1994). Identification of a hormonally regulated protein tyrosine phosphatase associated with bone and testicular differentiation. *Journal of Biological Chemistry*.

[15] Dacquin R, Mee PJ, Kawaguchi J (2004). Knock-in of nuclear localised *β*-galactosidase reveals that the tyrosine phosphatase Ptprv is specifically expressed in cells of the bone collar. *Developmental Dynamics*.

[16] Ferron M, Hinoi E, Karsenty G, Ducy P (2008). Osteocalcin differentially regulates *β* cell and adipocyte gene expression and affects the development of metabolic diseases in wild-type mice. *Proceedings of the National Academy of Sciences of the United States of America*.

[17] Yoshikawa Y, Kode A, Xu L (2011). Genetic evidence points to an osteocalcin-independent influence of osteoblasts on energy metabolism. *Journal of Bone and Mineral Research*.

[18] Fulzele K, Clemens TL (2011). Novel functions for insulin in bone. *Bone*.

[19] Pun KK, Lau P, Ho PWM (1989). The characterization, regulation, and function of insulin receptors on osteoblast-like clonal osteosarcoma cell line. *Journal of Bone and Mineral Research*.

[20] Kream BE, Smith MD, Canalis E, Raisz LG (1985). Characterization of the effect of insulin on collagen synthesis in fetal rat bone. *Endocrinology*.

[21] Hahn TJ, Westbrook SL, Sullivan TL, Goodman WG, Halstead LR (1988). Glucose transport in osteoblast-enriched bone explants: characterization and insulin regulation. *Journal of Bone and Mineral Research*.

[22] Fulzele K, Riddle RC, DiGirolamo DJ (2010). Insulin receptor signaling in osteoblasts regulates postnatal bone acquisition and body composition. *Cell*.

[23] Merlotti D, Gennari L, Dotta F, Lauro D, Nuti R (2010). Mechanisms of impaired bone strength in type 1 and 2 diabetes. *Nutrition, Metabolism and Cardiovascular Diseases*.

[24] Blüher M, Michael MD, Peroni OD (2002). Adipose tissue selective insulin receptor knockout protects against obesity and obesity-related glucose intolerance. *Developmental Cell*.

[25] Brüning JC, Michael MD, Winnay JN (1998). A muscle-specific insulin receptor knockout exhibits features of the metabolic syndrome of NIDDM without altering glucose tolerance. *Molecular Cell*.

[26] Ferron M, Wei J, Yoshizawa T (2010). Insulin signaling in osteoblasts integrates bone remodeling and energy metabolism. *Cell*.

[27] Scimeca JC, Franchi A, Trojani C (2000). The gene encoding the mouse homologue of the human osteoclast-specific 116-kDa V-ATPase subunit bears a deletion in osteosclerotic (oc/oc) mutants. *Bone*.

[28] Ferron M, McKee MD, Levine RL, Ducy P, Karsenty G (2012). Intermittent injections of osteocalcin improve glucose metabolism and prevent type 2 diabetes in mice. *Bone*.

[29] Brennan-Speranza TC, Henneicke H, Gasparini SJ (2012). Osteoblasts mediate the adverse effects of glucocorticoids on fuel metabolism. *The Journal of Clinical Investigation*.

[30] Cousin W, Courseaux A, Ladoux A, Dani C, Peraldi P (2004). Cloning of hOST-PTP: the only example of a protein-tyrosine-phosphatase the function of which has been lost between rodent and human. *Biochemical and Biophysical Research Communications*.

[31] Confavreux CB, Borel O, Lee F (2012). Osteoid osteoma is an osteocalcinoma affecting glucose metabolism. *Osteoporosis International*.

[32] Schwetz V, Pieber T, Obermayer-Pietsch B (2012). The endocrine role of the skeleton: background and clinical evidence. *European Journal of Endocrinology*.

[33] Im JA, Yu BP, Jeon JY, Kim SH (2008). Relationship between osteocalcin and glucose metabolism in postmenopausal women. *Clinica Chimica Acta*.

[34] Pittas AG, Harris SS, Eliades M, Stark P, Dawson-Hughes B (2009). Association between serum osteocalcin and markers of metabolic phenotype. *The Journal of Clinical Endocrinology & Metabolism*.

[35] Kindblom JM, Ohlsson C, Ljunggren O (2009). Plasma osteocalcin is inversely related to fat mass and plasma glucose in elderly Swedish men. *Journal of Bone and Mineral Research*.

[36] García-Martín A, Cortés-Berdonces M, Luque-Fernández I, Rozas-Moreno P, Quesada-Charneco M, Muñoz-Torres M (2011). Osteocalcin as a marker of metabolic risk in healthy postmenopausal women. *Menopause*.

[37] Hwang YC, Jeong IK, Ahn KJ, Chung HY (2011). Circulating osteocalcin level is associated with improved glucose tolerance, insulin secretion and sensitivity independent of the plasma adiponectin level. *Osteoporosis International*.

[38] Weiler HA, Lowe J, Krahn J, Leslie WD (2012). Osteocalcin and vitamin D status are inversely associated with homeostatic model assessment of insulin resistance in Canadian aboriginal and white women: the first nations bone health study. *The Journal of Nutritional Biochemistry*.

[39] Lee SW, Jo HH, Kim MR, You YO, Kim JH (2012). Association between obesity, metabolic risks and serum osteocalcin level in postmenopausal women. *Gynecological Endocrinology*.

[40] Gravenstein KS, Napora JK, Short RG (2011). Cross-sectional evidence of a signaling pathway from bone homeostasis to glucose metabolism. *The Journal of Clinical Endocrinology & Metabolism*.

[41] Ngarmukos C, Chailurkit LO, Chanprasertyothin S, Hengprasith B, Sritara P, Ongphiphadhanakul B (2012). A reduced serum level of total osteocalcin in men predicts the development of diabetes in a long-term follow-up cohort. *Clinical Endocrinology*.

[42] Kanazawa I, Yamaguchi T, Yamamoto M (2009). Serum osteocalcin level is associated with glucose metabolism and atherosclerosis parameters in type 2 diabetes mellitus. *The Journal of Clinical Endocrinology & Metabolism*.

[43] Yeap BB, Chubb SAP, Flicker L (2011). Reduced serum total osteocalcin is associated with metabolic syndrome in older men via waist circumference, hyperglycemia, and triglyceride levels. *European Journal of Endocrinology*.

[44] Saleem U, Mosley TH, Kullo IJ (2010). Serum osteocalcin is associated with measures of insulin resistance, adipokine levels, and the presence of metabolic syndrome. *Arteriosclerosis, Thrombosis, and Vascular Biology*.

[45] Kanazawa I, Yamaguchi T, Tada Y, Yamauchi M, Yano S, Sugimoto T (2011). Serum osteocalcin level is positively associated with insulin sensitivity and secretion in patients with type 2 diabetes. *Bone*.

[46] Bao Y, Zhou M, Lu Z (2011). Serum levels of osteocalcin are inversely associated with the metabolic syndrome and the severity of coronary artery disease in Chinese men. *Clinical Endocrinology*.

[47] Iglesias P, Arrieta F, Piñera M (2011). Serum concentrations of osteocalcin, procollagen type 1 N-terminal propeptide and *β*-CrossLaps in obese subjects with varying degrees of glucose tolerance. *Clinical Endocrinology*.

[48] Tan A, Gao Y, Yang X (2011). Low serum osteocalcin level is a potential marker for metabolic syndrome: results from a Chinese male population survey. *Metabolism*.

[49] Oosterwerff MM, van Schoor NM, Lips P, Eekhoff EM (2012). Osteocalcin as a predictor of the metabolic syndrome in older persons: a population-based study. *Clinical Endocrinology*.

[50] Yamashita T, Okano K, Tsuruta Y, Akiba T, Nitta K (2012). Serum osteocalcin levels are useful as a predictor of cardiovascular events in maintenance hemodialysis patients. *International Urology and Nephrology*.

[51] Pennisi P, Signorelli SS, Riccobene S (2004). Low bone density and abnormal bone turnover in patients with atherosclerosis of peripheral vessels. *Osteoporosis International*.

[52] Confavreux CB, Szulc P, Casey R (2013). Higher serum osteocalcin is associated with lower abdominal aortic calcification progression and longer 10-year survival in elderly men of the MINOS cohort. *The Journal of Clinical Endocrinology & Metabolism*.

[53] Reyes-Garcia R, Rozas-Moreno P, Jimenez-Moleon JJ (2012). Relationship between serum levels of osteocalcin and atherosclerotic disease in type 2 diabetes. *Diabetes & Metabolism*.

[54] Kapustin AN, Shanahan CM (2011). Osteocalcin: a novel vascular metabolic and osteoinductive factor?. *Arteriosclerosis, Thrombosis, and Vascular Biology*.

[55] Yeap BB, Chubb SAP, Flicker L (2011). Associations of total osteocalcin with all-cause and cardiovascular mortality in older men: the health in men study. *Osteoporosis International*.

[56] Flammer AJ, Gössl M, Widmer RJ (2012). Osteocalcin positive CD_133+_/CD_34-_/KDR+ progenitor cells as an independent marker for unstable atherosclerosis. *European Heart Journal*.

[57] Hwang YC, Jeong IK, Ahn KJ, Chung HY (2009). The uncarboxylated form of osteocalcin is associated with improved glucose tolerance and enhanced *β*-cell function in middle-aged male subjects. *Diabetes/Metabolism Research and Reviews*.

[58] Shea MK, Gundberg CM, Meigs JB (2009). *γ*-carboxylation of osteocalcin and insulin resistance in older men and women. *The American Journal of Clinical Nutrition*.

[59] Kanazawa I, Yamaguchi T, Yamauchi M (2011). Serum undercarboxylated osteocalcin was inversely associated with plasma glucose level and fat mass in type 2 diabetes mellitus. *Osteoporosis International*.

[60] Choi HJ, Yu J, Choi H (2011). Vitamin K2 supplementation improves insulin sensitivity via osteocalcin metabolism: a placebo-controlled trial. *Diabetes Care*.

[61] Pollock NK, Bernard PJ, Gower BA (2011). Lower uncarboxylated osteocalcin concentrations in children with prediabetes is associated with *β*-cell function. *Journal of Clinical Endocrinology and Metabolism*.

[62] Iki M, Tamaki J, Fujita Y (2012). Serum undercarboxylated osteocalcin levels are inversely associated with glycemic status and insulin resistance in an elderly Japanese male population: fujiwara-kyo osteoporosis risk in men (FORMEN) study. *Osteoporosis International*.

[63] Polgreen LE, Jacobs DR, Nathan BM, Steinberger J, Moran A, Sinaiko AR (2012). Association of osteocalcin with obesity, insulin resistance, and cardiovascular risk factors in young adults. *Obesity*.

[64] Bulló M, Moreno-Navarrete JM, Fernández-Real JM, Salas-Salvadó J (2012). Total and undercarboxylated osteocalcin predict changes in insulin sensitivity and *β* cell function in elderly men at high cardiovascular risk. *The American Journal of Clinical Nutrition*.

[65] Okuno S, Ishimura E, Tsuboniwa N (2012). Significant inverse relationship between serum undercarboxylated osteocalcin and glycemic control in maintenance hemodialysis patients. *Osteoporosis International*.

[66] Gundberg CM, Nieman SD, Abrams S, Rosen H (1998). Vitamin K status and bone health: an analysis of methods for determination of undercarboxylated osteocalcin. *Journal of Clinical Endocrinology and Metabolism*.

[67] Gundberg CM, Lian JB, Booth SL Vitamin K-dependent carboxylation of osteocalcin: friend or foe? 2012. *Advances in Nutrition*.

[68] Maïmoun L, Sultan C (2009). Effect of physical activity on calcium homeostasis and calciotropic hormones: a review. *Calcified Tissue International*.

[69] Levinger I, Zebaze R, Jerums G, Hare DL, Selig S, Seeman E (2011). The effect of acute exercise on undercarboxylated osteocalcin in obese men. *Osteoporosis International*.

[70] Fernández-Real JM, Izquierdo M, Ortega F (2009). The relationship of serum osteocalcin concentration to insulin secretion, sensitivity, and disposal with hypocaloric diet and resistance training. *Journal of Clinical Endocrinology and Metabolism*.

[71] Mokuda S, Okuda Y, Onishi M (2011). Post-menopausal women with rheumatoid arthritis who are treated with raloxifene or alendronate or glucocorticoids have lower serum undercarboxylated osteocalcin levels. *Journal of Endocrinological Investigation*.

[72] Simm PJ, Johannesen J, Briody J (2011). Zoledronic acid improves bone mineral density, reduces bone turnover and improves skeletal architecture over 2 years of treatment in children with secondary osteoporosis. *Bone*.

[73] Vestergaard P (2011). Risk of newly diagnosed type 2 diabetes is reduced in users of alendronate. *Calcified Tissue International*.

[74] Schwartz AV, Schafer AL, Grey A (2013). Effects of antiresorptive therapies on glucose metabolism: results from the FIT, HORIZON-PFT and FREEDOM trials. *Journal of Bone and Mineral Research*.

[75] Schafer AL, Sellmeyer DE, Schwartz AV (2011). Change in undercarboxylated osteocalcin is associated with changes in body weight, fat mass, and adiponectin: parathyroid hormone (1-84) or alendronate therapy in postmenopausal women with osteoporosis (the PaTH study). *The Journal of Clinical Endocrinology & Metabolism*.

[76] Forouhi NG, Ye Z, Rickard AP (2012). Circulating 25-hydroxyvitamin D concentration and the risk of type 2 diabetes: results from the European Prospective Investigation into Cancer (EPIC)-Norfolk cohort and updated meta-analysis of prospective studies. *Diabetologia*.

[77] Wolden-Kirk H, Overbergh L, Christesen HT, Brusgaard K, Mathieu C (2011). Vitamin D and diabetes: its importance for *β* cell and immune function. *Molecular and Cellular Endocrinology*.

[78] Oury F, Sumara G, Sumara O (2011). Endocrine regulation of male fertility by the skeleton. *Cell*.

[79] Kirmani S, Atkinson EJ, Melton 3rd LJ, Riggs BL, Amin S, Khosla S (2011). Relationship of testosterone and osteocalcin levels during growth. *Journal of Bone and Mineral Research*.

[80] Kanazawa I, Tanaka K, Ogawa N, Yamauchi M, Yamaguchi T, Sugimoto T (20122013). Undercarboxylated osteocalcin is positively associated with free testosterone in male patients with type 2 diabetes mellitus. *Osteoporosis International*.

[81] Hannemann A, Breer S, Wallaschofski H (2013). Osteocalcin is associated with testosterone in the general population and selected patients with bone disorders. *Andrology*.

[82] Seeman E (2002). Pathogenesis of bone fragility in women and men. *The Lancet*.

[83] Corona G, Monami M, Rastrelli G (2011). Type 2 diabetes mellitus and testosterone: a meta-analysis study. *International Journal of Andrology*.

